# A phenomenological study of female dance majors from single-parent families after psychological counseling by a counselor

**DOI:** 10.3389/fpsyg.2025.1518824

**Published:** 2025-08-01

**Authors:** Shuo Chen, Jing Zuo

**Affiliations:** School of Sport and Art, Shandong Sport University, Jinan, China

**Keywords:** female college students, interpersonal relationships, personal internal issues, insomnia, internet addiction, lifestyle habits

## Abstract

**Introduction:**

Single-parent families have recently become more common attributable to the increasing divorce rates in China. This has increased the prevalence of major psychological problems. Chinese tertiary students express their personal and developmental needs to school counselors as the key personnel in universities. Counselors provide standard social guidance to help with their psychological development and improvement. Herein, we conducted qualitative research with five female dance majors from single-parent families through in-depth interviews. Specifically, we sought to explore the impact of psychological counseling by counselors on the well-being of college students from single-parent families.

**Method:**

This qualitative research was primarily based on in-depth interviews for five female dance majors from single-parent families.

**Results:**

Eleven meaning units were identified, which were organized into five themes and two fundamental aspects, capturing the psychological counseling experiences of participants with their counselors. The five themes included interpersonal relationship concerns, personal internal issues, external symptoms of issues, changes in interpersonal relationships and internal issues, and persistence of personal habits. Furthermore, the counseling experience was largely defined by changes in individuals’ internal struggles and their external behaviors.

**Discussion:**

Our findings highlight the significance of psychological counseling counselors, the psychological problems affecting female college students from single-parent families, and the need for long-term interventions, given the persistent nature of personal habits such populations develop over time. Although this study did not specifically explore the psychological problems of students from single-parent families, it offers practical insights, which could guide future research in the realm of psychological counseling of such populations.

## 1 Introduction

Higher education enrollment in China has been steadily increasing. According to the People’s Republic of China’s Ministry of Education’s 2022 National Education Development Statistical Bulletin (released in 2023) (Ministry of Education of the People’s Republic of China, 2023), there are 3,013 higher education institutions in China. Among them, 1,239 are regular undergraduate institutions (including 164 independent colleges), one more compared to the previous year. Furthermore, there are 32 undergraduate vocational schools and 1,489 higher vocational (specialized) schools, a total of three more compared to the previous year. Moreover, 46.55 million students are currently enrolled in various Chinese higher education institutions, an increase of 2.25 million compared to the previous year. Additionally, the gross enrollment rate in Chinese higher education institutions is 59.6%, an increase of 1.8% compared to the previous year. It is also noteworthy that the average sizes of regular undergraduate schools, undergraduate vocational schools, and higher vocational (specialized) schools are currently 16,793, 19,487, and 10,168 students, respectively. Furthermore, regular undergraduate admissions have totaled 4.6794 million, an increase of 233,400 (5.25%) compared to the previous year. Additionally, there are 866,200 admissions to undergraduate programs in specialized schools. Furthermore, the total number of students admitted to various schools is 19.6564 million, an increase of 725,400 (3.83%) compared to the previous year. Moreover, graduates have totaled 4.7157 million, an increase of 434,700 (10.15%) compared to the previous year. It is also noteworthy that there are currently 764 private higher education institutions in China, accounting for 25.36% of the total number of higher education institutions nationwide. Among them, 390, 22, and 350, are regular undergraduate institutions, undergraduate vocational schools, and higher vocational (specialized) schools, respectively. These numbers indicate the continuous expansion of higher education learning in China, underscoring the need to strengthen students’ mental health education.

One of the biggest challenges new college students confront is not academic or employment-related, but rather interpersonal relationships. College students often develop psychological barriers attributable to macro factors and subjective perceptions, emotions, and personality, among other psychological deviations (Chen, 2015). According to the “Regulations on the Construction of Mentor Teams in General Higher Education Institutions” report, college counselors should fulfill eight main responsibilities, including “helping students cultivate a good moral character, frequently engaging students in heart-to-heart conversations, and providing targeted assistance to help students overcome specific issues related to academic success, career choices, friendships, and healthy living, thus improving their ideological understanding and spiritual realm” (Yuan and Gao, 2017). Reports such as “Opinions on Strengthening the Construction of Mentor and Class Teacher Teams in Higher Education Institutions,” “Regulations on the Construction of Mentor Teams in General Higher Education Institutions,” and “Professional Competency Standards for Counselors in Higher Education Institutions (Interim)” explicitly require counselors, as key players in college students’ ideological and political education, to frequently engage them in heart-to-heart conversations (Gao and Wang, 2024). Zhu (2021) reported that psychological counseling is crucial in promoting college students’ mental health, enhancing student identification, and building teacher-student trust, which collectively contribute to the effectiveness of heart-to-heart conversations. Moreover, an effective heart-to-heart conversation between students and counselors is crucial to the students’ personal development, as well as the counselors’ career growth (Li, 2020).

According to data from the People’s Republic of China’s Ministry of Civil Affairs, 2.879 million divorces were registered in 2022, an increase of 1.4% compared to the previous year (Ministry of Civil Affairs of the People’s Republic of China, 2023). In 2023, 2.593 million couples registered for divorce, and in 2024, this number rose slightly to 2.621 million (Ministry of Civil Affairs of the People’s Republic of China, 2025). Due to the annual increase in divorce rates in China, the number of college students from single-parent families is gradually increasing (Li, 2021). Compared to their counterparts from two-parent families, students from single-parent families are more prone to psychological issues such as introversion, low self-esteem, sensitivity, extremism, and higher incidences of emotional instability and psychological challenges (Wang et al., 2021). Furthermore, although such students crave social interaction and emotional recognition, they tend to be shy and passive in interpersonal relationships (Li, 2018), hindering their abilities to create good social networks (Dai, 2019). Based on this, this study explores the role of counselors in providing psychological support, grounded in their professional responsibilities. It aims to alleviate the psychological challenges faced by college students from single-parent families and emphasizes the importance of promoting their mental well-being through effective counseling interventions.

In recent years, with the continued advancement of mental health education in schools, the SCL-90 has become a widely used assessment tool for evaluating the mental health status of college and secondary school students (Liu and Zhang, 2004). The SCL-90 self-rating scale comprises 10 dimensions: Somatization, Obsessive-Compulsive, Interpersonal Sensitivity, Depression, Anxiety, Hostility, Phobia, Paranoia, Psychoticism, and additional symptoms (Hansen et al., 1953). The 5-point rating scale of the CL-90 indicates that a score above 3 suggests the subject is in a suboptimal psychological state or experiencing serious mental health issues (Cheng and Lu, 2011).

With gender as a stratification criterion, females were found to have a higher average total psychological stress score (182.5745) than males (179.9651). Female scores for somatization (1.0685), anxiety (1.0556), fear (1.0647), and psychoticism (1.0689) were all higher than those of males, indicating that the former generally experiences higher psychological stress incidences than the latter (Sun, 2024). Additionally, an analysis of factors influencing the overall mental health of vocational college students and undergraduate students indicates that male students generally exhibit better mental health than their female counterparts, regardless of academic level. This disparity may be associated with the inherent personality traits and temperamental differences, as females are often described as more emotionally sensitive, reflective, and emotionally delicate. During interpersonal and romantic interactions, they tend to invest more emotional energy and experience more complex emotional responses, which can impact emotional stability and psychological regulation (Yu and Zhao, 2012).

Based on surveys conducted at undergraduate and junior colleges, Liu (2018) reported that undergraduates at newly established universities exhibited generally weaker mental health than vocational students, somewhat reflecting the former’s poorer adaptability (as a minority group) to university life and their unique group characteristics. Furthermore, Tang (2012) highlighted significant variations in stress-coping strategies between graduating vocational students and undergraduates. While vocational students scored higher on mature stress coping strategies, undergraduates scored higher on immature coping strategies, indicating that in psychological crises, vocational and undergraduate students tend to employ mature and immature responses, respectively. In this regard, while developing psychological health education plans for all college students, it is essential to tailor certain aspects to the personality traits and mental health statuses of both undergraduates and vocational students, with special emphasis on improving undergraduate students’ mental health and personality development.

Psychological issues have been established to be quite significant among art students. Herein, we sought to elucidate the psychological challenges dance majors face. In a study by Li et al. (2024), it was deduced that dance majors distinctly differentiate among perceived competence, confidence, and self-efficacy. substantial psychological pressure among many art students in universities, potentially leading to numerous mental health issues that could hinder their healthy development and even cause severe problems such as depression, sensitivity, and suicidal tendencies (Yan et al., 2010).

Approximately 20% of university art students suffer from psychological disorders, with an increasing proportion taking leaves of absence or dropping out due to mental health problems (Wang, 2009). Furthermore, these students’ mental health levels are significantly lower than the national average, with female students experiencing more psychological issues than their male counterparts (Lin et al., 2012). In this regard, it is noteworthy that the mental health of university art students has recently garnered widespread attention. In addition to enhancing the understanding of the relationship between mental health status and personality traits, research on the mental health of art students could also aid educators in providing targeted educational services and resources (Xu, 2002; Wang, 2009; Yan et al., 2010; Lin et al., 2012; Bai, 2013).

Herein, we conducted qualitative research focusing primarily on female dance majors through in-depth interviews. This study presents several innovative features: it specifically targets female undergraduate students, investigates the psychological effects associated with a single-parent family background within this population, and employs a counselor-centered framework. This approach contributes a novel theoretical perspective for developing targeted mental health interventions for students from single-parent families. Our findings could offer valuable insights from both the educational and psychological perspectives. In addition to aiding counselors in conducting psychological counseling, our findings could also provide research directions for countermeasures aimed at promoting personal growth and studying the psychology of single-parent family students.

## 2 Materials and methods

### 2.1 Participants

As the mentor system in Chinese higher education institutions developed, the concept of counselors with the primary responsibility of guiding student ideological and political development gained more traction (Su, 2019). In September 2017, the newly revised “Regulations on the Construction of Mentor Teams in General Higher Education Institutions” further clarified counselors’ responsibilities, covering nine aspects: Ideological and theoretical education and value guidance, construction of party and league organizations and classes, academic style construction, daily student affairs management, psychological health education and counseling, online ideological and political education, response to campus crisis events, career planning and employment and entrepreneurship guidance, and theoretical and practical research (Zhang et al., 2022). According to research, counselors in Chinese higher education institutions hold a unique position (Wang and Zhang, 2008). Su (2019) reported that in the course of China’s higher education development, “counselors” have evolved into a special occupational group. They are key faculty members with a unique political identity, embodying the traits of traditional educators who “impart knowledge and resolve doubts” while also fulfilling certain practical functions related to student affairs management. Counselors primarily rely on party and league organizations, as well as class platforms to educate their audience on ideals and beliefs, select, cultivate, and motivate student leaders, clearly understand student development needs, care for student growth, and implement standardized and socialized education, all of which objectively promote student development and improvement.

Counselors in higher education institutions must undertake the dual role of teachers and practitioners in the realms of ideological and political education. As moral practice role models, counselors should guide students in developing correct values, achieving an internalized impact on students’ education and development through the practical outcomes of ideological and political education (Liang, 2020). Counselors should also adapt to changes in social consciousness among college students resulting from material developments and guide students in developing proper worldviews, values, and perspectives on life (Hu, 2021). Therefore, to more effectively address psychological challenges faced by students and support their health development, we involved counselors with extensive professional experience and consistent history of conducting psychological consultations as key participants.

Sufficiency and appropriateness are two vital sampling criteria in qualitative research (Morse and Field, 1995). This study involved seven participants, which is in accordance with Creswell’s (2015) recommendation of having 5–25 appropriate participants to ensure “sufficiency”. Initially, two students served as pilot participants to test the feasibility of the interview process. Based on the challenges observed during these initial sessions, adjustments were made to improve the protocol. Subsequently, 5 students from single-parent families were formally enrolled as participants in the main study. The characteristics of the study participants are summarized in Table 1.

**TABLE 1:** Participants’ general characteristics.

Counselors	Family situation	Question	Age	Gender
S1	Single-parent family, living with the mother, with little contact with the father.	Lack of attention and self-control; Insomnia; Introverted personality	18	Female
S2	Single-parent family, living with the mother, has had no contact with the father since the parents’ divorce.	Internet addiction; Introverted and timid personality; Difficulty interacting with the opposite sex; Expressing emotions by throwing things.	18	Female
S3	Single-parent family, living with the mother.	Difficulty in communicating effectively; Introverted personality; Limited interaction with the opposite sex	18	Female
S4	Single-parent family, living with the mother and a half-brother from the father’s side. Has had no contact with the father for many years, and was raised by the maternal grandmother.	Addiction to online gaming, using late-night gaming sessions to release emotions; Introverted personality; Fear of engaging in nurturing communication	18	Female
S5	Single-parent family, living with the mother and younger brother, with strained relationship and minimal contact with the father.	Feelings of inferiority; Loneliness, sadness, feeling out of place; Insomnia; Difficulty in interacting with the opposite sex; Lack of security	18	Female

In this study, seven female undergraduate students majoring in dance performance at Shandong Sport University were enrolled in a voluntary approach. The single-parent family background—which was defined as living with a single parent for ≥5 years before the age of 18—was determined through questionnaires and interviews following the Classification Criteria for Family Structure model. Moreover, the Symptom Checklist 90 (SCL-90) was employed to assess the psychological states of the participants. Table 2 presents the SCL-90 test results, along with the corresponding comprehensive evaluation outcomes. Data from the semi-structured interviews were systematically integrated with the study findings to determine the presence and classify the specific types of psychological problems. Recruitments were conducted face-to-face and all participants were comprehensively informed about the research, its content, and purpose, among other relevant details. To protect confidentiality and personal privacy concerns, all participants are anonymized, with the individual names replaced by S.

**TABLE 2:** SCL-90 test results and comprehensive evaluation conclusions.

Counselors	SCL-90 depression factor score	SCL-90 anxiety factor score	Comprehensive evaluation conclusion
S1	2.5	2.2	Mild depressive and anxiety tendencies
S2	2.8	2.6	Mild depression with moderate anxiety tendencies
S3	3.1	2.3	Moderate depression with mild anxiety tendencies
S4	2.2	2.0	Slight depressive and anxiety tendencies
S5	2.7	2.4	Mild depression with slight anxiety tendencies

The first participant (S1) was in junior high school when her parents divorced. She is currently living with her mother and has minimal contact with her father. Her hobbies include sleeping and binge-watching dramas. She finds her current school life to be different from what she imagined, leading to some kind of discomfort. She has suffered from a lack of attention and self-discipline since her parents’ divorce and rarely initiates any communication with her parents. She also has a poor relationship with her college roommates and prefers solitude. It is also noteworthy that she has experienced insomnia, restlessness, and intrusive thoughts since high school.

The second participant (S2) has been living with her mother and lost contact with her father after her parents’ divorce. She is currently addicted to internet browsing and enjoys reading online novels. She argues about everything, which often puts her in conflict with her mother. She also has a poor relationship with her roommates and avoids contact with the opposite sex. Additionally, she is introverted and expresses her emotions by throwing things when upset.

The third participant (S3) currently lives with her mother. She feels that there is a lack of understanding and communication between her and her mother. She also has an average relationship with roommates and struggles to communicate. Furthermore, she is introverted and avoids strangers for fear that they could harbor ill intentions. Additionally, she rarely interacts with the opposite sex and mostly listens to music with headphones when in a bad mood.

The fourth participant (S4) currently lives with her mother and half-brother. She was raised by her grandmother and rarely communicates with her father. She is also addicted to online games, introverted, and prefers solitude. Additionally, she lacks the confidence to communicate (attributable to her parents’ divorce), avoids contact with the outside world, and stays up late playing games to vent emotions.

The final participant (S5) currently lives with her mother and younger brother. Her parents divorced after her college entrance examination. She has witnessed frequent parental quarrels since childhood and feels that she is a burden to her mother. Furthermore, she has low self-esteem and dislikes initiating communication with classmates. Additionally, she often suffers from insomnia and poor sleep quality. It is also noteworthy that she rarely communicates with the opposite sex and lacks a sense of security.

### 2.2 Data collection

This study was largely based on questioning and counseling sessions, which were divided into three parts: personal background pre-counseling, personal situation during counseling, and personal situation post-counseling. Each counseling session lasted ∼30 min. The questions asked during the three sessions were generally consistent, covering four main areas: current life situation, mentor counseling experience, current academic situation, and future plans.

The study began in December 2022 during which initial ideas were generated. From February to June 2023, the primary focus goals were topic selection, finding relevant literature, establishing theoretical backgrounds, and writing. The study officially commenced in September 2023, starting with participant selection and interview guideline development. The selected participants underwent two preliminary interviews and the interview guidelines were revised and adjusted based on the results. From March 2024, the participants underwent formal interviews and the collected data were organized. From April to June 2024, the results were analyzed and summarized.

### 2.3 Interview process

#### 2.3.1 Preliminary interview (January 11–February 23, 2024)

Initially, experimental interviews were conducted with two female students. The original plan was for each interview to last 1–1.5 h. However, due to the extended duration, both students struggled to stay focused during the conversations. They also felt burdened and uncomfortable when recalling past issues, further hindering deeper discussions.

#### 2.3.2 Formal interview (March 2024)

Based on the issues encountered during the preliminary interviews, we made some adjustments to the subsequent interview plans. For instance, the interview duration was reduced to ∼30 min each time. Furthermore, one-on-one interviews were repeated three times. Additionally, the questionnaire content was modified regarding sentence structure and wording. We also selected specific interview locations per participants’ preferences to ensure a relaxed and engaged atmosphere during the interviews. Moreover, question sequences were altered based on the direction of the conversations and additional relevant questions were incorporated based on individual circumstances. With participants’ consent, the entire interview was recorded both audibly and in writing.

### 2.4 Material analysis

Herein, we sought to explore the experiences and insights of counselors in addressing psychological issues female dance performance majors from single-parent families face. In qualitative research, data analysis often involves a cyclic process of collection, classification, restructuring, interpretation, and drawing conclusions (Yin, 2013). Measures to ensure the reliability of the findings were as follows:

First, in “collection,” a significant amount of literature related to the topic was reviewed and analyzed. Second, in “classification,” the contents of the recorded interviews during in-depth discussions were transcribed and annotated and then categorized into higher- and lower-level themes for coding. Third, in “restructuring,” the segmented data from the classification stage were structured. Fourth, in “interpretation,” experiences and behavioral needs of research participants were described and explanations were provided based on previous research. Fifth, in “drawing conclusions,” the overall content was reviewed again and the significance and implications of the study were reviewed using appropriate terminologies and concepts.

To avoid bias, the triangulation approach was employed to analyze the data from multiple perspectives based on factual data on mentor psychological counseling. This study’s validity was established through the analysis of the data coded in the initial round. To ensure reliability, the process involved reconfirming the transcribed content of participants’ interviews. Based on the validated data, we supplemented and refined the transcribed materials for a comprehensive description of participants’ experiences, elucidating underlying intentions and meanings. Finally, the data were contextualized and categorized for improved understanding and interpretation by readers.

### 2.5 Research ethics

The Ethics Committee of Sports Science at Shandong Sport University approved this study, which was conducted from September 2023 to November 2024. Before the initial interviews, all participants received detailed research descriptions and agreements approved by the aforementioned Ethics Committee, after which they provided informed consent.

### 2.6 Research credibility

It is critical to ensure the effectiveness and credibility of the study when collecting and analyzing interview data. Lincoln and Guba (1985) proposed credibility as a primary criterion for evaluating qualitative research, arguing that it covers four key attributes, including trustworthiness, transferability, dependability, and confirmability. Table 3 summarizes the definitions of these attributes and the techniques that were used to achieve them.

**TABLE 3:** Credibility: categories and techniques.

Category	Definition	Techniques used in this study
Credibility	The suitability of participants’ perspectives and researchers’ representation of them	Triangulation of data was achieved through at least three face-to-face interviews per week with each participant, followed by the transmission of interview records to participants for member checking and validation.
Transferability	The generalizability of the study results	Conducted three in-depth, detailed interviews with each participant, provided detailed descriptions of participants’ growth experiences, and analyzed their background and environment.
Dependability	Consistency and reliability of the research process and findings	Provided a clear and transparent description of the research process, recorded all interviews, promptly transcribed them, and asked the participants to verify the accuracy and reliability of the content.
Confirmability	The extent to which the interpretation and findings can be confirmed by others	Maintained a scientific attitude toward reproducing phenomena in research materials and interview data, engaged in prolonged self-reflection before and after each interview; and consulted an expert in the relevant field to check for potential biases in data collection and analysis, as well as provided suggestions for interview techniques.

## 3 Research findings

Herein, 11 units of meaning related to psychological issues were identified and aggregated into five themes, detailed in this section and Tables 4, 5. Participants’ psychological issues and their etiologies after their parents divorced are also elaborated, including the identified units of meaning, themes, and supporting text excerpts.

**TABLE 4:** Personal psychological state before counseling: themes, meaning units, and references.

Themes	Meaning unit	References
1. Interpersonal relationship issues.	1) Social phobia.	“I believe that not staying in contact with my father will affect me because I lack experience in communicating with the opposite sex and do not know how to express my thoughts to them. Sometimes, I even struggle to express myself with same-sex friends and do not know how to initiate conversations with others. I have no plans to date, partly because my mother’s failed marriage has had a significant impact on me.” (S2, 2024.03.04)
		“I do not know how to initiate conversations with others. During my time in college, I have no intention of finding a boyfriend. I enjoy being alone and feel that boys generally have bad intentions, so I do not really want to interact with them.” (S3, 2024.03.04)
		“I rarely interact with the opposite sex. Perhaps I am somewhat self-conscious and do not know how to communicate with them. I also lack a sense of security.” (S5, 2024.03.07)
	2) Communication barriers.	“I have some conflicts with my roommates. I prefer to be alone and spend time in a quiet place alone. I do not initiate communication with my parents because I feel they might be more at ease without hearing from me. I already feel a lot of pressure now.” (S1, 2024.03.03)
		“I do not currently share things that happen at school with my mom because she contradicts me no matter what I say. Now I just think about it but do not act on them.” (S2, 2024.03.04)
		“I do not want my father to contact me proactively.” (S4, 2024.03.04)
		“I feel very unfamiliar with the school environment right now because I come from a single-parent family. I am afraid that my classmates might look down on me or make fun of me, so I do not want to interact with them too much.” (S5, 2024.03.07)
2. Personal internal issues.	3) High psychological stress.	“Ever since my parents divorced, my self-control and focus have not been as good as they used to be. I feel like my parents might be more at ease without me. I feel that I may be a huge burden on them, and it’s difficult to communicate normally.” (S1, 2024.03.03)
		“I have conflicts with my roommates, as well as with my parents.” (S2, 2024.03.04)
		“I sometimes throw pens or similar objects to vent my emotions.” (S3, 2024.03.04)
		“After my parents’ divorce, I feel particularly inferior, different from other classmates, and afraid of being laughed at by them. I mainly follow my mother, feeling like I have caused her a great burden.” (S5, 2024.03.07)
	4) Social inhibition.	“I dislike crowded places. I do not want teachers and classmates to know about my situation. I do not want others to understand my affairs.” (S1, 2024.03.03)
		“I do not know how to communicate with the senior sisters. I feel timid, nervous, and prone to making mistakes. I feel unsuitable for the work in the discipline inspection department.” (S2, 2024.03.04)
		“I tend to internalize things when I encounter issues, meaning I’m unwilling to talk to others about them.” (S3, 2024.03.04)
		“I do not know how to get along with classmates and prefer to be alone. I do not interact much with the boys in my class, possibly because of my father. I lack the desire to engage with others.” (S4, 2024.03.04)
3. External symptoms of the problem.	5) Insomnia disorder.	“Every evening, my mood worsens. Along with being influenced by external factors, I may become restless and uneasy, overthinking things, which makes me feel increasingly uncomfortable. As a result, I start experiencing insomnia and can’t fall asleep. I’ve had this problem since high school, but back then, I thought it was due to academic pressure. I did not expect it to persist into college and continue to this day.” (S1, 2024.03.03)
		“I have insomnia. I feel very tired early in the day, but once I lie down, I either do not fall asleep or I keep waking up throughout the night. The next day, I still feel extremely tired and sleepy, as if I have not slept at all. My sleep quality is poor.” (S5, 2024.03.07)
	6) Internet addiction.	“I usually enjoy browsing the internet and reading online novels.” (S2, 2024.03.04)
		“I really enjoy playing games, I’m particularly addicted to it. Without any special hobbies or talents, I just like playing games. I use gaming as a way to vent my inner unhappiness.” (S4, 2024.03.04)

**TABLE 5:** Personal psychological state after receiving counseling: themes, meaning units, and references.

Themes	Meaning unit	References
4. Changes in interpersonal relationships and inner issues	7) There has been some relief in opposite-sex interactions, but significant issues still remain	“I am increasingly able to interact with boys, not avoiding conversations as before. Although there’s some improvement, I am still hesitant due to the fear that boys might deceive or harm me.” (S4, 2024.03.04)
	8) Communication has been alleviated	“My relationship with my roommates has improved, and the pressure in both life and studies has decreased. I can communicate with my mom and seek her advice on matters. These changes are due to the frequent communication and counseling sessions in receive from the mentor. The mentor’s help accounts for about 80% of my improvement. I am grateful for the counselor’s assistance.” (S2, 2024.03.04)
		“I still do not interact much with my father, but the biggest impact it has on me is that I understand my mother better. I want to help her with some of the things I can do.” (S3, 2024.03.04)
	9) Improvement in psychological stress	“I now meet with the mentor about twice a week, with each session lasting between half an hour to forty minutes. During our conversations, the mentor helps me brainstorm solutions to my problems based on my concerns. They also provide encouragement and support in many aspects, guiding me out of the darkness and improving my mood.” (S1, 2024.03.03)
		“The mentor tells us about various things, including professional matters and stories about world champions, which has made me a bit more confident.” (S3, 2024.03.04)
		“Sometimes when I am not feeling good, I run or take a walk around the playground to clear my mind a bit. If I’m feeling particularly down and really want to get out, I watch a movie or have a meal alone.” (S5, 2024.03.07)
	10) Transitioning from introverted and reclusive to more outgoing	“Through communication with the mentor, there have been changes in various aspects of my life. Firstly, my academic performance, daily life, and communication with my family have improved. Relationships are now more harmonious than before, and I no longer blame myself or others unreasonably.” (S2, 2024.03.04)
		“The mentor helped me integrate into the class, adapt to campus life, and blend in with everyone. The biggest help the mentor provided for me is that it made my personality more outgoing, lively, and confident, allowing me to interact more with the outside world. Deep down, I still aspire to be a more outgoing and a lively person.” (S4, 2024.03.04)
5. Personal habits remain unchanged	11) Internet addiction is closely related to insomnia issues and has not seen significant relief	“I still experience sleep problems to some extent. When I am feeling down, I still experience insomnia at night. I toss and turn, overthinking unnecessary things.” (S1, 2024.03.03)
		“Nowadays, when I am feeling restless, I do not vent by throwing things like I used to. However, I still often stay up late watching anime or similar things. It’s become a habit.” (S2, 2024.03.04)
		“I still often play games, but I go to bed earlier now. There’s been some improvement, and I’ve started taking naps in the afternoon.” (S4, 2024.03.04)
		“I still experience insomnia. It is the same as before, where I feel tired early but sometimes cannot fall asleep. I still wake up frequently during the night, and my sleep quality is not very good.” (S5, 2024.03.07)

Two basic aspects of the experiences of female dance majors from single-parent families were identified in their interactions with counselors during psychological counseling sessions: Changes in personal internal issues and changes in personal external issues. Table 6 presents the details of participants’ structural experiences during counseling sessions with counselors.

**TABLE 6:** Experiential structure of counseling sessions for female university students majoring in dance in single-parent families.

Meaning unit	Category	Essential aspects
Interpersonal communication barriers.	Interpersonal relationship issues	Changes in personal external issues
Communication barriers.		
Insomnia disorder.	External symptoms of the problem	
Internet addiction.		
Internet addiction is closely related to insomnia issues and has not seen significant relief.	Personal habits remain unchanged	
High psychological stress.	Personal internal issues	Personal internal issue changes
Introverted and solitary personality.		
Opposite-sex interactions have seen some relief but significant issues still remain.	Changes in interpersonal relationships and inner issues.	
Communication has been alleviated.		
Psychological stress has improved.		
Introversion is beginning to adjust from being reclusive.		

Theme 1: Interpersonal Relationship Issues

This theme primarily focuses on the impacts on female dance majors after their parents divorced, particularly regarding interpersonal relationships with classmates and friends. Students from single-parent families generally exhibited resistance and avoidance traits when facing internal issues in their daily lives, as well as poor communication skills due to a lack of or limited communication with their fathers.

Unit of Meaning 1: Social Phobia

Social phobia, also known as an anxiety disorder, is defined as excessive fear of and avoidance of social situations or interpersonal interactions. It is characterized by the fear of being scrutinized, negatively judged, or rejected by others, as well as the worry of self-mockery or embarrassed in social settings, leading to active avoidance of social contexts, including parties, public speaking, and workplace communication. In their daily study and lives, students from single-parent families tended to avert normal communication or interactions with classmates and friends, especially with friends of the opposite sex. Notably, due to the nature of the dance major, which requires communication and practice with partners, these students’ psychological issues may affect their communication and professional practice.

“I think not contacting my father for a long time will have an impact on me because I lack communication skills with the opposite sex. I do not know how to express my thoughts to the opposite sex. Sometimes, I cannot express myself well, even with same-sex friends. Although our major requires mixed doubles in practical exams and cooperation with male partners, but because I have had no physical contact with males for a long time, it can be difficult during lifts, as there is a hugging action, but I am resistant, so it wastes a lot of time. I do not plan to have a boyfriend during my university years. One of the reasons is that my mother’s marriage failure has had a big impact on me.” (S2, 2024.03.04)

“I do not know how to communicate actively with others. When I meet strangers, I feel like they have ill intentions. Most of the time, I have these thoughts during interactions with female friends. I do not have plans to find a boyfriend during my university years. I like being alone. I feel that boys are more malicious, so I do not want to interact with them.” (S3, 2024.03.04)

“I hardly communicate with the opposite sex. Mainly because of my parents, I feel there are no topics. Maybe I’m also quite inferior, do not know how to communicate with the opposite sex, and lack a sense of security.” (S5, 2024.03.07)

Unit of Meaning 2: Communication Barriers

Communication barriers are defects or obstacles that significantly limit the ability to achieve proper communication, impairing information transmission or reception. Specifically, it comprises obstacles within specific communication channels, such as language, speech, hearing, and cognition, and may be caused by physiological, psychological, or environmental factors. Children from single-parent families often conflict with their parents; hence, they cannot communicate with them effectively. In addition to showing rebellious attitudes toward their parents, these students felt inferior when facing unfamiliar friends; hence, they lacked critical communication skills and struggled to make new friends.

“I have experience conflicts with my roommates because I am not familiar with them. I prefer to be alone. My mother usually contacts me actively, and my dad also contacts me, but I do not take the initiative to communicate with them because I feel it would be more relaxing without their involvement. University life is not particularly suitable or enjoyable for me because it is very different from what I imagined. I already feel a lot of pressure now.” (S1, 2024.03.03)

“I currently do not share some of the things happening at school with my mom because no matter what I say, she always contradicts me. Since I had many extracurricular classes on weekends when I was young, she always said no whenever I wanted to do something fun on weekends. Therefore, now I just think about it but do not actually do it.” (S2, 2024.03.04)

“I do not contact my father anymore. My mother is a more open-minded person. I might have wanted to contact him when I was young, but as I grew up, I no longer wanted to. Initially, when I heard classmates sharing about their fathers, I might have felt uncomfortable psychologically. It took about half a year or more to adapt. I also do not want my father to contact me actively.” (S4, 2024.03.04)

“I feel very unfamiliar with the school environment now and with classmates and teachers around me. I feel very lonely and uncomfortable because I’ve never come this far away from home to study alone. I am afraid that if my classmates know about my situation, they will isolate me because I come from a single-parent family. I’m afraid they’ll look down on me or make fun of me, so I feel quite uncomfortable and inferior. I feel like I’m less that others and do not want to interact too much with my classmates or let them know too much about me.” (S5, 2024.03.07)

Theme 2: Personal Internal Issues

This theme relates to internal issues attributable to the fact that the participants were from single-parent families. While personal internal issues vary from person to person, we found that dance majors tended to experience high psychological stress and introverted personalities.

Unit of Meaning 3: High Psychological Stress

Parents’ divorce affected participants’ academic performance and mood. Furthermore, due to the lack of education and companionship from one parent, the participants’ self-discipline decreased, and they tended to enjoy being alone. They could also resort to throwing objects to relieve inner pressure.

“I initially found it difficult to cope with university life. Academic pressure has become overbearing since due to reduced self-discipline and attention following the divorcing of my parents. I feel like my parents would be more relaxed without me around. I feel like a huge burden to them, and I am not sure whether they expect or hope me to contact them regularly. Also, we do not have common topics during conversation, and we have different opinions, which leads to conflicts and makes normal communication impossible.” (S1, 2024.03.03)

“I experience significant academic pressure. This creates conflicts with my roommates and parents, damaging our relationship. I experience challenges associated with dormitory hygiene and sleep issues.” (S2, 2024.03.04)

“I feel upset at home and sometimes throw pens or other things to vent my emotions. The most rebellious thing I do is disobedience to my mother. I do not follow her instructions. I just pretend to making sure she does not know.” (S3, 2024.03.04)

“After my parents’ divorce, my life has changed, and I feel my family is incomplete. I used to go out with my parents, but now I only go out with my mom. I feel particularly inferior and different from other classmates, and I’m afraid they will laugh at me. My mother pays for my living expenses, and I depend mainly on her. I feel that I am imposing a huge burden on her, therefore, I try to save money whenever I can because I feel my mother is working very hard, this makes me very uncomfortable.” (S5, 2024.03.07)

Unit of Meaning 4: **Social Inhibition**

Social inhibition is defined as excessive display of caution, hesitation, or behavioral restraint in social situations, accompanied by the fear of exploring actions deemed socially unacceptable. Due to the incomplete nature of single-parent families, most participants feel inferior and unwilling to communicate with others, creating an introverted and reclusive personalities.

“I dislike crowded places. I feel more relaxed and efficient when I do things alone. Sometimes, I do not dare to say what I want to say, especially private thoughts and issues. I do not want teachers and classmates to know about my situation or understand my affairs.” (S1, 2024.03.03)

“I joined the student union’s discipline department before, but after a week, I found that my personality and various aspects were not suitable for this job, so I quit. Because the discipline department checks rooms every night and conducts morning exercises, I am not outgoing and do not know how to communicate with senior sisters. I feel timid, nervous, and prone to mistakes, and thus, unsuitable for the discipline department’s work.” (S2, 2024.03.04)

“I listen to music through earphones when in a bad mood and avoid talking to friends. I have lived like this since high school because there was a lot of academic pressure in high school, and I had limited time to study cultural subjects for the arts exam. When the college entrance examination was approaching, I just buried myself in studying every day and did not talk much. I found it annoying, and there were also some family reasons. I tend to internalize things when I encounter problems and do not want to share with others.” (S3, 2024.03.04)

“I am still adapting to university life. Since my first arrival in university, I have always felt uncomfortable due to the different routine from that in high school. I do not know how to interact with classmates and prefer to be alone. Initially, I did not talk much with my roommates or others, I was too afraid to participate in conversations. For me, it takes time to open up and communicate with others. I also avoid much interaction with male students in my class, maybe because of my father’s influence. If I have to participate in a group discussion or a collaborative project with male students from different classes, it stresses me out psychologically. I might speak less and just want it to exit the discussion before it ends.” (S4, 2024.03.04)

Theme 3: External Symptoms of Problems

This theme relates to personal problem symptoms associated with single-parent family background, primarily manifesting as insomnia and addiction to the internet. Insomnia is generally defined as a subjective experience that comprises the feeling of dissatisfaction with the duration, quality, or both, of sleep, thereby affecting an individual’s daytime social functioning. Insomnia presents as the difficulty of falling asleep (taking >30 min to fall asleep), sleep maintenance disorders (experiencing ≥2 awakenings throughout the night), early awakening, reduced sleep quality, and decreased total sleep time (typically < 6 h), accompanied by daytime dysfunction (Sleep Disorders Group of the Chinese Society of Neurology, 2012). Internet addiction should not be narrowly classified as a clinical disorder given that it involves complex multifactorial interactions that extend beyond traditional disease frameworks. Excessive internet use among adolescents is often associated with societal issues involving family upbringing, learning, and peer influence. Therefore, joint efforts from multiple parties are necessary to improve and resolve these problems (Xinhua News and Agency, 2018). According to the Core Information and Interpretation of Health Education for Chinese Adolescents (2018 Edition), internet addiction refers to uncontrollable impulses toward internet use without the influence of addictive substances. This addictive behavior manifests as significant impairment in academic, occupational, and social functions due to excessive internet use (National Health Commission of the People’s Republic of China, 2018). Based on this definition, we performed pre-study participant evaluation using methods such as self-reporting and observation of living habits. The students enrolled in this study met the diagnostic criteria for insomnia and internet addiction.

Unit of Meaning 5: Insomnia

Participants reported experiencing insomnia due to factors such as overthinking and high stress levels.

“I have conflicts with my roommate because I cannot sleep at night. My roommate wakes up early in the morning, which disturbs my sleep. Because of this conflict, we do not communication. Every night, my mood worsens, and I become restless and anxious due to external influences. I start overthinking, which makes me even more uncomfortable, leading to insomnia. I had this problem in high school too, but I thought it was because of academic pressure. I did not anticipate that it will persist to university.” (S1, 2024.03.03)

“I experience insomnia. I feel tired early in the evening, but I cannot fall asleep when I lie down. Sometimes I sleep for a while and then wake up. The next day, I still felt tired, as if I had not slept at all. My sleep quality is poor.” (S5, 2024.03.07)

Unit of Meaning 6: Internet Addiction

Internet addiction arises from isolating oneself and seeking relief from stress and emotions through activities like online gaming and reading web novels.

“I usually like to read web novels online. When I was in second or third grade, my academic performance was not good, and my mom was often away. Therefore, she bought me a tablet for studying. From then on, after finishing my homework, I would use the tablet to read comics and watch animations. This behavior continued until junior high. During that time, my mother was frequently traveling on business trips, but in junior high, her trips became less frequent, and she was available more often.” (S2, 2024.03.04)

“I enjoy playing video games, especially in high school. It helps me pass the time. I use gaming to vent my sadness.” (S4, 2024.03.04)

Theme 4: Changes in Interpersonal Relationships and Inner Issues

After the counseling sessions with a mentor, students adjusted their interpersonal relationships and communication, encountering problems without evasion. This increased their communication with classmates, friends, and parents.

Unit of Meaning 7: Some Relief in Romantic Relationships, but Significant Issues Persist

Due to the long-term impact of single-parent families, although there has been some improvement in romantic relationships, significant issues remain, such as no plans for marriage.

“I have slowly started to interact with boys, not avoiding conversations as much. However, there’s still some resistance in my heart. I am still influenced by my mother and am somewhat afraid of boys deceiving me and causing me harm.” (S4, 2024.03.04)

Unit of Meaning 8: Improvement in Communication

The students mentioned that they were able to resolve many issues due to frequent meetings with the mentor. Their relationships with roommates, classmates, and interaction with parents improved.

“My relationship with my roommate has improved, and the stress in life and studies has decreased. I can now ask my mother for her opinion, even though she still disagrees with me. She’s beginning to understand the reasons behind our desires as young people, although she may not verbally support me, she expresses it through actions. The frequent communication and counseling by the mentor has significantly changed my life.” (S2, 2024.03.04)

“My contact with my father has not increased significantly, but the counseling has improved the understanding with my mom and I want to share some of her burdens.” (S3, 2024.03.04)

Unit of Meaning 9: Improvement in Psychological Stress

Through counseling and encouragement from the mentor, the students expressed that their perspectives on problems have widened, allowing them to emerge from depression, and their mood has improved.

“I now meet with the mentor about twice a week, and each session lasts between 30 min and 40 min. During the sessions, the mentor helps me solve my problems through encouragement and guidance, which improves my mood.” (S1, 2024.03.03)

“The mentor discusses various topics, including professional matters and stories about world champions, which has improved by confidence.” (S3, 2024.03.04)

“Sometimes when I am feeling sad, I go for a run or take a walk on the playground to clear my mind. If I feel particularly depressed and want to go out, I often watch a movie or have dinner alone.” (S5, 2024.03.07)

## 4 Discussion

The purpose of this study was to expand the current understanding of the role of mentor psychological counseling and how to it can be applied more efficiently. Data were collected through in-depth interviews for 5 female university students majoring in dance performance from single-parent families. The data analysis identified 11 meanings, 5 themes, and 2 basic aspects of mentor psychological counseling. In-depth examination of the psychological impact of counselor intervention on female college students majoring in dance performance from single-parent families identified strong associations among various fundamental factors and emergent themes. Counselors were included in the meticulous assessment of personal external and internal issues of the participants, formulation and implementation of targeted counseling strategies, thereby significantly contributing to emergent themes.

### 4.1 Two basic aspects of the experience of counselor psychological counseling among female university students majoring in dance performance from single-parent families

The first fundamental aspect targeted modification of personal external challenges, in terms of five categories of external issues that participants identified as requiring urgent adjustment during the psychological counseling process. The results showed that female college students majoring in dance performance from single-parent family backgrounds faced significant obstacles in interpersonal interaction and collaboration in their daily study and life due to changes in family structure. This demonstrates a nuanced tension with the conventional interpretation of post-traumatic growth theory. Linley and Joseph (2005) proposed that individuals with a high level of post-traumatic growth exhibits greater psychological resilience and an optimistic tendency. However, this theory does not comprehensively account for the unique dilemmas of adolescents from single-parent families in the context of the superimposition of intense training in art majors and emotional deprivation. The distinctive competitive environment and heightened emotional expression demands in art colleges make this population particularly vulnerable to exhibiting emotional communication difficulties during interpersonal interactions. These findings underscore the differential influence of family environment on the social adaptability of individuals in specialized professional fields. Regarding the external symptoms of psychological distress, we found that insomnia and internet addiction were prevalent among students from single-parent families. These findings are consistent with the proposition by Jiang’s (2024) that college students experiencing interpersonal problems often turn to mobile networks as coping mechanisms, with some exhibiting a tendency to retreat into the virtual environments to avoid real-life challenges. However, in our study, we further examined the behavioral characteristics of art students. It was observed that due to pressure associated with long-term physical training and performance anxiety, dance performance majors are more inclined to seek emotional compensation and self-recognition through the internet in the absence of family support. Notably, in terms of the reshape of personal habits, this study resolves some of the limitations in the existing research. For instance, majority of previous studies focused on the intervention effects of psychological counseling on cognition and emotions. In contrast, the present study show, for the first time, that long-term formation of behavioral inertia by art students (such as self-enclosed creative habits and fragmented time management patterns) exhibit significant poor response to psychological intervention, providing a new perspective that will potentially improve the design of personalized intervention programs. By focusing on the specific group of dance performance majors, we address the limitation of psychological intervention for students from single-parent families pursuing performance art. However, our results must be interpreted with caution given the relatively limited number of psychological counseling interventions employed in this study, which may have affected the effectiveness of the counseling program and the generalizability of the research results. Moreover, the involvement of few counseling sessions may not have been sufficient to completely address the deeply ingrained behavioral inertia of the participants. Some students may have prematurely ended the intervention before fully adapting to the new behavioral patterns, resulting in the incomplete manifestation of behavioral change effects. Additionally, the brief duration of the intervention limited the observation of long-term behavioral habit formation, making it difficult to fully evaluate the enduring impact of psychological counseling on the modification of personal habits. Therefore, further in-depth investigations into the dynamic process of behavioral habit reshaping among art students by extending the intervention cycle, increasing the frequency of counseling sessions, and incorporating long-term follow-up surveys are advocated. These measures will significantly improve the design and facilitate the implementation of personalized intervention programs.

Although previous studies primarily employed universal samples, in-depth interviews were conducted, which showed the association between professional characteristics (such as body language expression training and stage performance pressure) and family trauma. This not only extends the theoretical framework of counselors’ psychological counseling, but also offers empirical support for the development of a “professional–family–psychological” triadic intervention model.

The second fundamental aspect relates to the transformation of personal internal issues, exploring the underlying psychological dilemmas manifested by participants during counseling, as well as the dynamic progression of their interpersonal relationships and internal emotional states. Using motivation theory, Sheng (2002) found that, although intrinsic motivation is not the only form of human motivation, it significantly influences individual growth and shapes the behavior. Based on this theory, we observed that the reconstruction of the internal psychological state of female college students from single-parent families may largely represent a process of activating and restructuring their intrinsic motivation system. In the context of personal internal issues identified in Theme 2, results showed that the psychological trauma caused by parental divorce not only induced introverted behaviors and social withdrawal but contributed to the activation of deep-seated psychological stressors. Accumulating evidence indicates that children from single-parent families are likely to develop depression and anxiety due to their passive attitudes and fear of social adaptation (Park et al., 2014). This study further supports this assertion by demonstrating the unique manifestations within the context of art majors: the special requirements of dance performance majors for emotional expression and body language strongly conflict with the students internal emotional withdrawal, exacerbating the contradictions in their self-perception. For example, some participants developed pronounced self-repressive tendencies due to fear of revealing their true emotions during stage performances. Within the domain of interpersonal relationship changes and internal psychological conflicts identified in Theme 4, this study advances beyond the scope of conventional psychological intervention research by integrating innovative theoretical perspectives and methodological strategies. In practical application, counselors adopt a dual intervention-monitoring strategy based on the framework of “emotional mapping-behavioral imitation”. Regarding the challenges related to heterosexual communication, role-playing is used to simulate stage interaction scenarios, transferring the physical coordination developed through dance training to real-life social interactions. In addressing psychological stress, counselors integrate dance-specific breathing regulation techniques with mindfulness-based therapy, enabling students to translate their pre-performance tension management strategies into effective tools for daily emotional regulation. This model integrates professional characteristics with psychological interventions to promote qualitative provide intervention strategies that resolve psychological distress in the context of romantic relationships and interpersonal communication, confirming the unique value of the synergistic effect of professional backgrounds and psychological counseling. This study not only shows that psychological counseling can improve internal psychological state of students from single-parent families but also provides a new approach for enhancing the implementation of psychological counseling strategies by revealing the underlying mechanism between art professional training and psychological interventions. In this regard, this study presents a more effective therapeutic approach compared to similar studies. The interactive influence between professional characteristics and psychological changes identified in this study lays an important theoretical foundation for the construction of a composite intervention model of based on the “art empowerment-psychological reconstruction” framework, thereby addressing the knowledge gap on the psychological intervention strategies for special professional groups.

### 4.2 Revisiting the research questions

The first research question examines the existence relatively severe psychological challenges among students before receiving psychological counseling. It is divided into three thematic areas: interpersonal relationship issues, personal intrinsic problems, and external symptomatic problems. Following parental divorce, the students experienced substantial changes to their development owing to the absence of timely psychological intervention. They manifested in six significant units of meaning: interpersonal communication obstacles, communication difficulties, high psychological stress, introverted and isolated personality traits, insomnia, and internet addiction.

The second research question aimed to explore the improvements observed in students’ interaction with the opposite sex, communication, stress management, and the adjustment of introverted personality traits following completion of counseling sessions with counselors. These positive outcomes will improve the participants’ growth in learning and daily life by enhancing their communication skills. However, due to the long-term formation of personal habits among the researchers, one significant unit of meaning related to internet addiction and insomnia did not improve significantly, which may require prolonged intervention.

The third research question explored the strategies which may be employed by counselors to help participants cultivate positive and optimistic attitudes toward learning and life, enabling them to engage in social activities optimally and avoid psychological disturbances, to positively transform their lives. The results indicated that counselors need to understand the students’ current psychological conditions and concerns from multiple perspectives before initiating interventions tailored to each individual. Moreover, they should exhibit patience and confidence in addressing students’ habitual problems, to improve the long-term and effective intervention.

All counselors involved in this study are board-certified with extensive professional experience in psychological counseling. Nevertheless, some limitations were observed, especially in resolving complex psychological issues, particularly those related to the long-term reshaping of behavioral habits. Therefore, we suggest that counselors need to strengthen their psychological counseling competencies. In the future, continuous professional training and participant follow-up should be adopted to improve the professionalism and effectiveness of the intervention implemented by counselors within the psychological counseling practice.

### 4.3 Implications, limitations, and future research

This study has important implications for improving the implementation of psychological counseling by counselors for students from single-parent families. Our findings underscore the importance of psychological counseling by counselors. Given the psychological conditions of participants, counselors should pay close attention to the tone, attitude, and sensitivity of their inquiries during the initial stages of counseling. They should strive to articulate issues and statements as clearly and candidly as possible to ensure participants’ understanding. Furthermore, counselors are advised to avoid seeking immediate results during counseling sessions, instead providing participants with sufficient time to overcome psychological barriers and reflect on their challenges. They should also encourage participants to engage in school-organized activities and offer support and care whenever feasible. Moreover, it is recommended to select a warm and tranquil counseling environment and consider playing soothing music to facilitate participant relaxation and enhance session outcomes.

The sample characteristics used in this study have significant limitations. The current research only selected five 18-year-old female college students majoring in dance performance from a single university as the sample. The extremely small sample size and highly homogeneous group have severely restricted the generalizability of the research results. With the continuous increase in the divorce rate, future related studies urgently need to significantly expand the sample size, including participants of different genders, majors, age groups, and from various regions and institutions. This will enhance the universality of the research conclusions. In addition, subsequent research should focus on the psychological status of students from divorced families during childhood, such as primary and junior high school stages. By advancing the psychological intervention strategies, psychological problems can be prevented at an early stage.

In the future, we plan to investigate the psychological impact of dance therapy on students from single-parent families. Moreover, we shall enroll a larger sample size to include more diverse participants, covering individuals from varying educational levels and geographical backgrounds. This approach will enhance the generalizability of research findings by enabling a more in-depth exploration of the effective psychological intervention strategies.

## 5 Conclusion

This was a phenomenological study on the experiences of five female university students majoring in dance performance from single-parent families who received psychological counseling with counselors. Through in-depth interviews, we collected data and analyzed five themes, addressing three research questions.

Firstly, before receiving counseling from counselors, students had relatively severe psychological issues, to the degree that affected by their normal learning and daily lives. Following the divorce of their parents and the resulting incomplete family structure, participants perceived changes in their relationships with friends and classmates, leading to feelings of psychological inferiority and introverted personalities. There were significant obstacles in their interpersonal communication, developing tendencies to avoid unfamiliar people and issues related to their own family, especially harboring resistance toward the opposite sex, lacking intentions for dating and marriage, and poor sense of security. Participants also experienced guilt when interacting with their parents, owing to the feeling that they might have partially contributed to their parents’ divorce, resulting in psychological stress. Due to the lack of timely intervention, these issues affected their studies and lives, causing challenges such as insomnia and internet addiction.

Secondly, after counseling by counselors, the students exhibited significant improvement in various aspects, including interpersonal relationships, communication, psychological stress, and personality adjustment. was Although there were minor improvements in personal habits, no significant change was observed. During the study period, additional three informal meetings were conducted together with the three formal psychological counseling sessions per week. These informal meetings aimed to monitor and assess the psychological dynamics of participants in real-time and promptly capture emotional fluctuations and behavioral changes. Notably, these informal interactions were not part of the traditional counseling sessions, instead, they were relaxed conversations involving active participation. For example, activities such as group campus walks and the review of basic dance exercises were organized as part of the intervention. This approach enabled the observation of psychological state of students in natural settings and timely detection of potential problems. Moreover, to effectively assist participants in addressing psychological issues, counselors conducted interviews with participants’ roommates and class leaders to gain additional insights into their current situations and existing problems, thereby facilitating accurate judgment formation. To accelerate the improvement, counseling sessions were accompanied by engaging simple activities such as volunteering and dance performances, which were aligned with the participants’ professional characteristics and the degree of social interaction. At the end of the study, participants’ interpersonal relationships, communication, and psychological stress exhibited significant improvement, enabling them to actively engage in communication with others, alleviate tensions in their relationships with their parents, and experience a positive future. However, in cases of insomnia and internet addiction, the effectiveness of intervention measures—such as sleep hygiene education and behavioral contracts for internet use—is relatively limited. This may be attributed to the chronic and complex nature of these problems. This finding suggests that for deeply ingrained behavioral patterns, long-term, continuous, and more targeted intervention strategies should be formulated.

Thirdly, mentor-led psychological counseling can facilitate the students to address psychological issues and issues encountered in their studies and lives. As role models for moral practice among university students, counselors should guide students to cultivate correct worldviews, outlooks on life, and values, as well as assist them in resolving psychological problems and life difficulties. Mentor-led psychological counseling may help students to identify their own issues and make corresponding adjustments through guidance and assistance. The students become empowered to better adapt to university and social life, with little conflicts between students and their families.

During psychological counseling, counselors should enhance their own psychological counseling abilities by acquiring professional psychology knowledge to improve their professionalism and effectiveness. Moreover, counselors should first understand the basic condition of the students so that they do not ask questions that may harm them. Tailored intervention plans should be developed in advance in line with the participants’ personal circumstances and combined with mentor’s work experience to ensure effective counseling. Furthermore, counselors should intervene depending on the participants’ personal problems and personality traits, and if necessary, communicate with participants’ parents in a timely manner. Finally, for issues such as personal habits that are not easily changed, counselors should be ready to provide long-term interventions using appropriate methods.

In conclusion, we performed an empirical analysis to explore the practical value and implementation strategies of psychological counseling provided by counselors to students majoring in dance from single-parent families. The results provide an important theoretical foundation that will guide future research. Nevertheless, future research need to explore more universally acceptable intervention models and employ extended longitudinal follow-up data to capture the long-term effects of the interventions.

## Data Availability

The original contributions presented in this study are included in this article/supplementary material, further inquiries can be directed to the corresponding author.
